# Crystal structure and Hirshfeld surface analysis of 3-[2-(3,5-di­methyl­phen­yl)hydrazinyl­idene]benzo­furan-2(3*H*)-one

**DOI:** 10.1107/S2056989021011749

**Published:** 2021-11-12

**Authors:** Zeliha Atioğlu, Mehmet Akkurt, Namiq Q. Shikhaliyev, Ulviyya F. Askerova, Sevinc H. Mukhtarova, Rizvan K. Askerov, Ajaya Bhattarai

**Affiliations:** a Department of Aircraft Electrics and Electronics, School of Applied Sciences, Cappadocia University, Mustafapaşa, 50420 Ürgüp, Nevşehir, Turkey; b Department of Physics, Faculty of Sciences, Erciyes University, 38039 Kayseri, Turkey; c Organic Chemistry Department, Baku State University, Z. Xalilov str. 23, Az, 1148 Baku, Azerbaijan; d Department of Chemistry, M.M.A.M.C (Tribhuvan University) Biratnagar, Nepal

**Keywords:** crystal structure, intra­molecular N—H⋯O inter­actions, C—H⋯π inter­actions, Hirshfeld surface analysis

## Abstract

In the title compound, C_16_H_14_N_2_O_2_, the 2,3-di­hydro-1-benzo­furan ring system is essentially planar and makes a dihedral angle of 3.69 (7)° with the di­methyl­phenyl ring. The mol­ecular conformation is stabilized by an intra­molecular N—H⋯O hydrogen bond with an *S*(6) ring motif. In the crystal, mol­ecules are connected by C—H⋯π and π–π stacking inter­actions, forming a layer lying parallel to the (11  ) plane. One methyl group is disordered over two orientations, with occupancies of 0.67 (4) and 0.33 (4). Hirshfeld surface analysis indicates that the most important contributions to the crystal packing are from H⋯H (51.2%), O⋯H/H⋯O (17.9%), C⋯H/H⋯C (15.2%) and C⋯C (8.1%) contacts.

## Abstract 2

In the crystal, mol­ecules are connected by C—H⋯π and π–π stacking inter­actions, forming a layer lying parallel to the (11 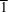 ) plane.

## Chemical context

Hydrazones have many applications in diverse areas, such as in optical data storage, as mol­ecular switches and anti­microbial agents, in non-linear optics, mol­ecular recognition, dye-sensitized solar cells, color-changing materials, catalysis, liquid crystals, *etc*., mainly because of the azo-to-hydrazo tautomerism/isomerism and the optical properties of –N=N– unit (Maharramov *et al.*, 2018 ▸; Ma *et al.*, 2020 ▸, 2021 ▸; Viswanathan *et al.*, 2019 ▸). Not only *E/Z* isomerization, but also azo-hydrazone tautomerism is important in organic and the coordination chemistry of hydrazone dyes (Ma *et al.*, 2017*a* ▸,*b* ▸; Mahmoudi *et al.*, 2017 ▸, 2018 ▸). The design of hydrazone dyes with electron donor or acceptor substituents has led to multidentante ligands, the corresponding coordination compounds of which have been applied effectively as catalysts in oxidation and C—C coupling reactions (Mahmudov *et al.*, 2013 ▸; Mizar *et al.*, 2012 ▸). Moreover, the functional properties of hydrazones or their metal complexes can be regulated by attaching functional groups to the =N—NH— unit (Gurbanov *et al.*, 2020*a* ▸,*b* ▸; Kopylovich *et al.*, 2011 ▸; Mahmudov *et al.*, 2020 ▸; Shixaliyev *et al.*, 2014 ▸). Thus, we have attached C=O groups and furan and aryl rings to the =N—NH— moiety, leading to a new hydrazone compound, (*Z*)-3-[2-(3,5-di­methyl­phen­yl)hydrazinyl­idene]benzo­furan-2(3*H*)-one, which can form inter­molecular inter­actions. 
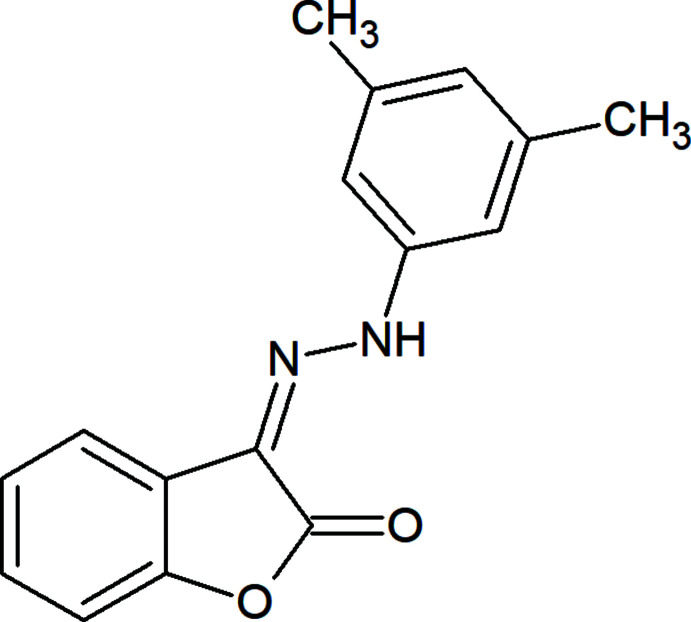


## Structural commentary

**Figure 1:**
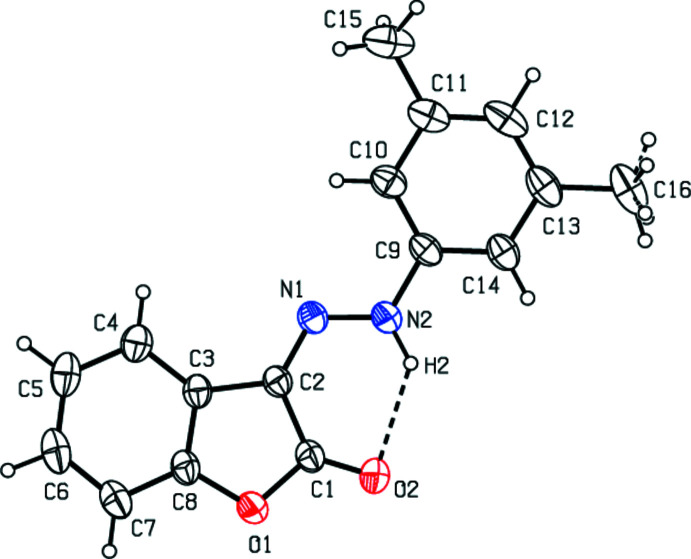
The mol­ecular structure of the title compound with displacement ellipsoids for the non-hydrogen atoms drawn at the 30% probability level.

The mol­ecular conformation of the title compound is stabilized by an intra­molecular N—H⋯O hydrogen bond (N2—H2⋯O2; Table 1 ▸) with an *S*(6) ring motif (Fig. 1 ▸). The 2,3-di­hydro-1-benzo­furan ring system (O1/C1–C8) of the title compound is essentially planar [maximum deviations = −0.031 (2) Å for C3 and 0.026 (2) Å for C6] and makes a dihedral angle of 3.69 (7)° with the di­methyl­phenyl C9–C14 ring. In the mol­ecule, the aromatic C9–C14 ring and the C=N—NH– unit are almost coplanar with a dihedral angle of 4.8 (8)° between them.

## Supra­molecular features

**Figure 2:**
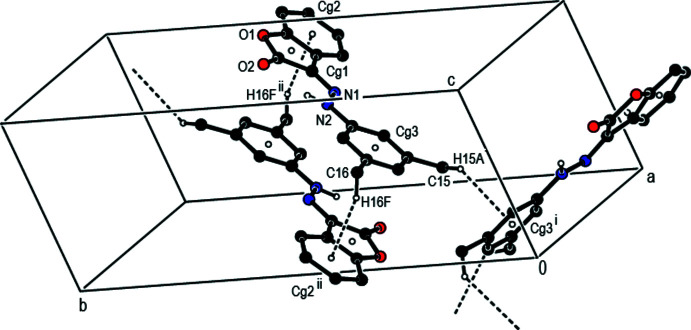
A partial packing view of the title compound, showing the C—H⋯π inter­actions (dashed lines). Only H atoms involved in the inter­actions and N-bound H atoms are shown for clarity [Symmetry codes: (i) *x*, −*y* − 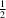 , *z* − 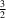 ; (ii) −*x* + 1, −*y* + 1, −*z* + 1.]

**Figure 3:**
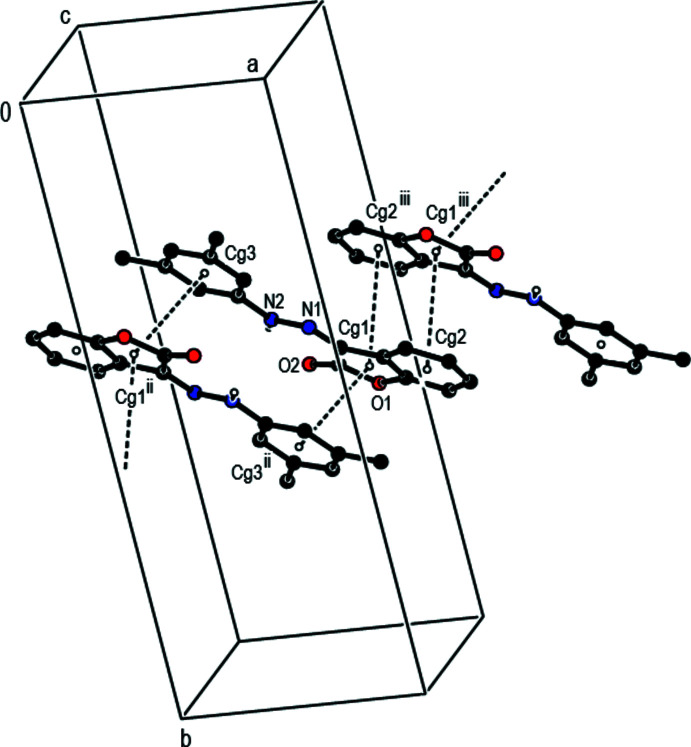
A partial packing view of the title compound, showing the π–π stacking inter­actions (dashed lines). Only N-bound H atoms are shown for clarity [Symmetry codes: (ii) −*x* + 1, −*y* + 1, −*z* + 1; (iii) 2 − *x*, 1 − *y*, 2 − *z*.]

**Figure 4:**
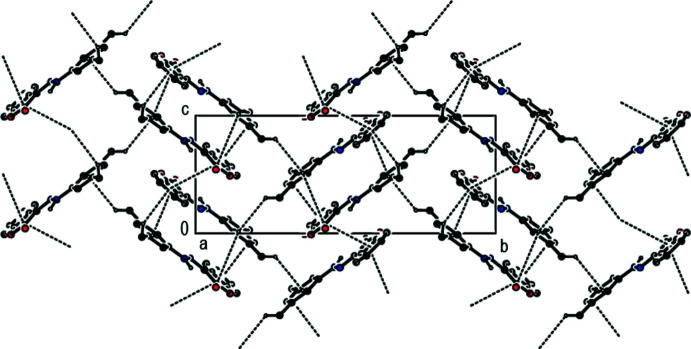
A packing diagram of the title compound viewed along the *a*-axis, showing C—H⋯π inter­actions (dashed lines). Only H atoms involved in the inter­actions and N-bound H atoms are shown for clarity.

In the crystal, mol­ecules are connected by C—H⋯π inter­actions [C15—H15*A*⋯*Cg*3^i^ and C16—H16*F*⋯*Cg*2^ii^; symmetry codes as given in Table 1 ▸; Fig. 2 ▸] and π–π stacking inter­actions [*Cg*1⋯*Cg*2^îii^ = 3.6227 (11) Å, slippage = 1.226 Å; *Cg*1⋯*Cg3* ^îi^ = 3.7128 (10) Å, slippage = 1.339 Å; Symmetry codes: (ii) −*x* + 1, −*y* + 1, −*z* + 1; (iii) −*x* + 2, −*y* + 1, −*z* + 2], where *Cg*1 and *Cg*2 are the centroids of the oxolane O1/C1–C3/C8 and benzene C3–C8 rings, respectively, of the 2,3-di­hydro-1-benzo­furan ring system while *Cg*3 is the centroid of the di­methyl­phenyl C9–C14 ring (Fig. 3 ▸). These inter­actions link the mol­ecules into a layer structure lying parallel to the (11 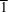 ) plane (Fig. 4 ▸).

## Hirshfeld surface analysis

**Figure 5:**
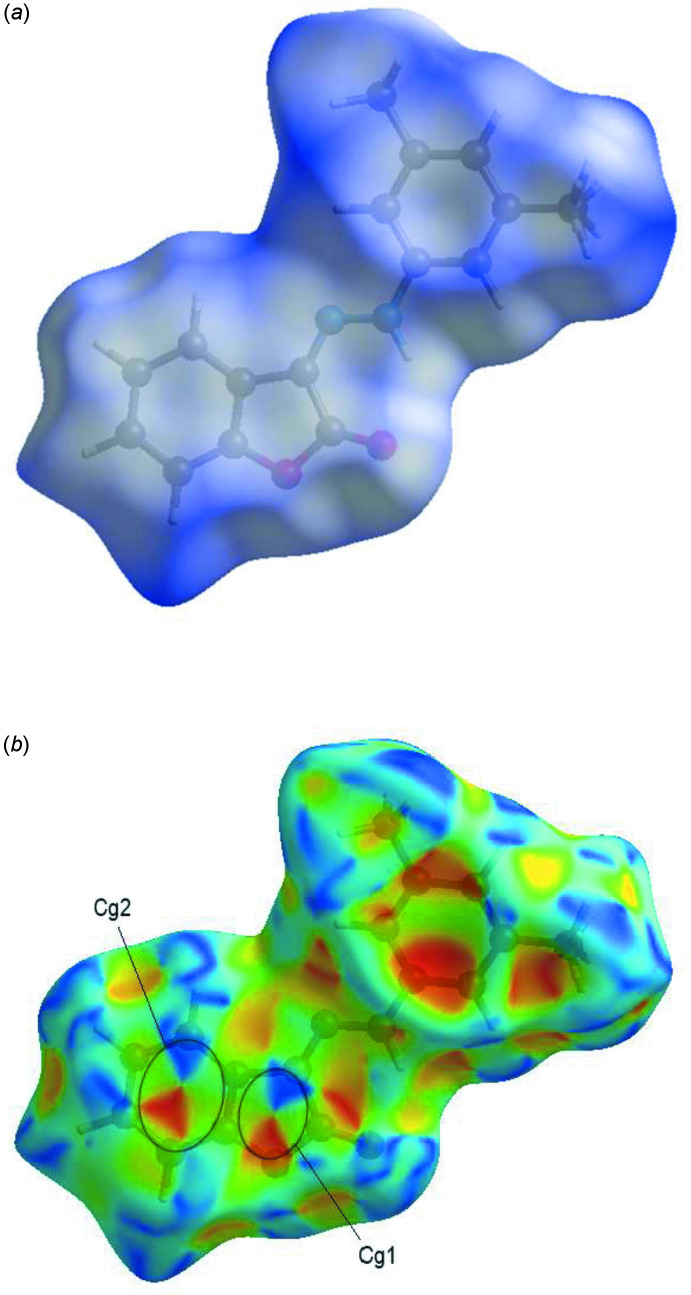
Hirshfeld surfaces of the title mol­ecule, (*a*) mapped with *d*_norm_ in the range −0.0001 to 1.5993 a.u. and (*b*) plotted over shape-index.

*Crystal Explorer*17 (Turner *et al.*, 2017 ▸) was used to perform a Hirshfeld surface analysis and generate the associated two-dimensional fingerprint plots, with a standard resolution of the three-dimensional *d*_norm_ surfaces plotted over a fixed color scale of −0.0001 (red) to 1.5993 (blue) a.u. (Fig. 5*a* ▸). All of the disordered H atoms of the C16 methyl group were taken into account together. The shape-index of the Hirshfeld surface is a tool to visualize the π–π stacking by the presence of adjacent red and blue triangles; if there are no adjacent red and/or blue triangles, then there are no π–π inter­actions. Fig. 5 ▸ *b* clearly indicates that there are π–π inter­actions in the title compound.

**Figure 6:**
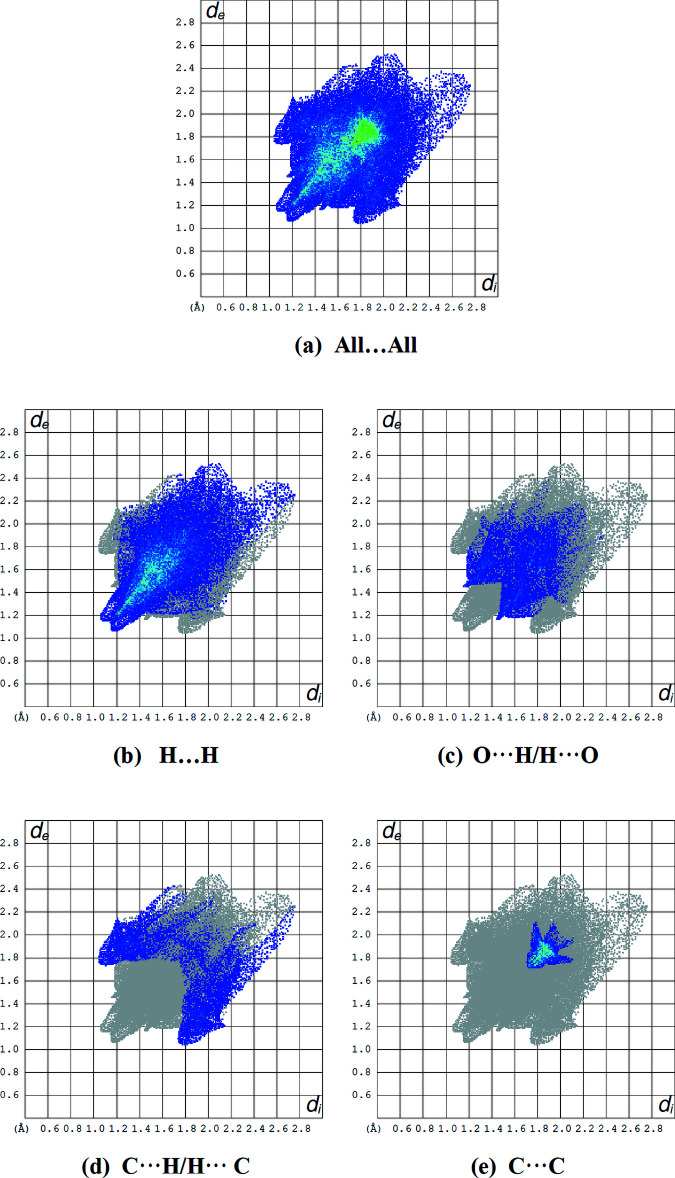
Fingerprint plots showing (*a*) all inter­molecular inter­actions and delineated into (*b*) H⋯H, (*c*) O⋯H/H⋯O, (*d*) C⋯H/H⋯C and (*e*) C⋯C contacts.

Two-dimensional fingerprint plots for the H⋯H, O⋯H/H⋯O, C⋯H/H⋯C and C⋯C contacts are presented in Fig. 6 ▸. H⋯H inter­actions, which are located in the middle region of the fingerprint plot, contribute the most to overall crystal packing, with 51.2% (Fig. 6*b* ▸). The O⋯H/H⋯O contacts contribute 17.9% (Fig. 6*c* ▸) to the Hirshfeld surface, while the C⋯H/H⋯C contacts contribute 15.2% (Fig. 6*d* ▸), resulting in a pair of distinctive wings. The C⋯C inter­actions account for 8.1% of the Hirshfeld surface. The percentage contributions to the Hirshfeld surface including other minor ones are summarized in Table 2 ▸.

## Database survey

A search of the Cambridge Crystallographic Database (CSD version 5.42, updated September 2021; Groom *et al.*, 2016 ▸) for the 1-benzo­furan-2(3*H*)-one unit gave 220 hits. Of these, the compound most similar to the title compound is 7-meth­oxy-3-(2-phenyl­hydrazinyl­idene)-1-benzo­furan-2(3*H*)-one, **I** (CSD refcode IBADIC; Atioğlu *et al.*, 2021 ▸). Four compounds reported by Oliveira *et al.* (2019 ▸) are closely related to the title compound, *viz*. 2-(4-nitro-1*H*-imidazol-1-yl)-*N*′-[1-(pyridin-2-yl)ethyl­idene]acetohydrazide, **II** (TODMEH), 2-(2-nitro-1*H*-imidazol-1-yl)-*N*′-[1-(pyridin-2-yl)ethyl­idene]acetohydrazide, **III** (TODMIL), 2-(4-nitro-1*H*-imidazol-1-yl)-*N*′-[phen­yl(pyridin-2-yl)methyl­idene]acetohydrazide, **IV** (TODMOR) and 2-(4-nitro-1*H*-imidazol-1-yl)-*N*′-[phen­yl(pyridin-2-yl)methyl­idene]acetohydrazide, **V** (TODMUX). Compound **I** crystallizes in the monoclinic space group *C*2/*c* with *Z* = 8. In the crystal of **I**, pairs of mol­ecules are linked into dimers by N—H⋯O hydrogen bonds, forming an 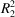 (12) ring motif, with the dimers stacked along the *a-*axis direction. These dimers are connected through π–π stacking inter­actions between the centroids of the benzene and furan rings of their 2,3-di­hydro-1-benzo­furan ring systems. Furthermore, there exists a C—H⋯π inter­action that consolidates the crystal packing. Compounds **II** and **IV** crystallize in the monoclinic space group *P*2_1_/*c* with *Z* = 4. Compound **III** crystallizes in the monoclinic space group *I*2/*a* with *Z* = 8 and **V** crystallizes in the triclinic space group *P* 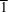 with *Z* = 2. Compound **VI** crystallizes in the monoclinic space group *P*2_1_/*c* with *Z* = 4. The *E* conformation in **II**, **III** and **V** is stabilized by a strong inter­molecular N—H⋯O inter­action. These inter­actions lead to the formation of dimeric structural arrangements. In the crystal of **IV**, an inter­molecular N—H⋯N hydrogen bond results in a helical chain structure along the *b*-axis direction. Non-classical inter­molecular C—H⋯N and C—H⋯O inter­actions are also observed in the crystals of **II**, **III**, **IV** and **V**.

## Synthesis and crystallization

(*Z*)-3-[2-(3,5-Di­methyl­phen­yl)hydrazinyl­idene]benzo­furan-2(3*H*)-one was synthesized according to the reported method (Shikhaliyev *et al.*, 2018 ▸, 2019 ▸). A 20 mL screw-neck vial was charged with DMSO (10 mL), (*E*)-2-{[2-(3,5-di­methyl­phen­yl)hydrazinyl­idene]meth­yl}phenol (240 mg, 1 mmol), tetra­methyl­ethylenedi­amine (TMEDA) (295 mg, 2.5 mmol), CuCl (2 mg, 0.02 mmol) and CCl_4_ (20 mmol, 10 equiv). After 1–3 h (until TLC analysis showed complete consumption of the corresponding Schiff base), the reaction mixture was poured into a 0.01 *M* solution of HCl (100 mL, pH 2–3), and extracted with di­chloro­methane (3 × 20 mL). The combined organic phase was washed with water (3 × 20 mL), brine (30 mL), dried over anhydrous Na_2_SO_4_ and concentrated *in vacuo* using a rotary evaporator. The residue was purified by column chromatography on silica gel using appropriate mixtures of hexane and di­chloro­methane (3/1–1/1). Then the substance was refluxed in methanol for 30 min, and left for evaporation. After three days, single crystals of the title compound suitable for X-ray analysis were obtained. Colorless solid (65%); m.p. 475 K. Analysis calculated for C_16_H_14_N_2_O_2_ (*M* = 266.30): C 72.17, H 5.30, N 10.52; found: C 72.13, H 5.26, N 10.48%. ^1^H NMR (300 MHz, CDCl_3_) *δ* 12.04 (1H, NH), 6.79–7.69 (7H, Ar), 2.37 (6H, 2CH_3_). ^13^C NMR (75 MHz, CDCl_3_) *δ* 160.47, 157.77, 134.91, 125.14, 124.12, 121.77, 121.56, 119.86, 118.21, 114.55, 108.16, 106.59, 16.85 and 16.52. ESI–MS: *m*/*z*: 267.23 [*M* + H]^+^.

## Refinement details

Crystal data, data collection and structure refinement details are summarized in Table 3 ▸. The amine H atom was located in a difference-Fourier map and refined freely [N2—H2 = 0.93 (2) Å]. All C-bound H atoms were placed at calculated positions using a riding model, with C—H = 0.93 or 0.96 Å, and with *U*_iso_(H) = 1.2 or 1.5*U*_eq_(C). The methyl group with the C16 atom attached to the atom C13 is disordered over two orientations, with occupancies of 0.67 (4) and 0.33 (4). Owing to poor agreement, nine reflections ( 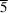 14 10, 7 13 0, 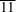 6 5, 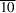 12 4, 11 1 0, 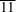 1 1, 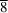 19 6, 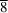 0 8 and 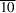 17 4) were omitted during the final refinement cycle.

## Supplementary Material

Crystal structure: contains datablock(s) I. DOI: 10.1107/S2056989021011749/is5559sup1.cif

Structure factors: contains datablock(s) I. DOI: 10.1107/S2056989021011749/is5559Isup2.hkl

Supporting information file. DOI: 10.1107/S2056989021011749/is5559Isup3.cml

CCDC reference: 1983650

Additional supporting information:  crystallographic information; 3D view; checkCIF report

## Figures and Tables

**Table 1:** Hydrogen-bond geometry (Å, °) *Cg*2 and *Cg*3 are the centroids of the C3–C8 and C9–C14 rings, respectively.

*D*—H⋯*A*	*D*—H	H⋯*A*	*D*⋯*A*	*D*—H⋯*A*
N2—H2⋯O2	0.93 (2)	2.12 (2)	2.840 (2)	133.8 (16)
C15—H15*A*⋯*Cg*3^i^	0.96	2.90	3.591 (3)	130
C16—H16*F*⋯*Cg*2^ii^	0.96	2.92	3.715 (3)	141

Symmetry codes: (i) x, -y-{\script{1\over 2}}, z-{\script{3\over 2}}; (ii) -x+1, -y+1, -z+1.

**Table 2:** Percentage contributions of inter­atomic contacts to the Hirshfeld surface of the title compound

Contact	Percentage contribution
H⋯H	51.2
O⋯H/H⋯O	17.9
C⋯H/H⋯C	15.2
C⋯C	8.1
N⋯C/C⋯N	4.2
N⋯H/H⋯N	1.9
O⋯C/C⋯O	0.9
N⋯N	0.6
O⋯O	0.1

**Table 3:** Experimental details

Crystal data	
Chemical formula	C_16_H_14_N_2_O_2_
*M*_r_	266.29
Crystal system, space group	Monoclinic, *P*2_1_/*c*
Temperature (K)	296
*a*, *b*, *c* (Å)	8.8644 (4), 19.9222 (8), 8.1736 (3)
β (°)	107.240 (1)
*V* (Å^3^)	1378.59 (10)
*Z*	4
Radiation type	Mo *K*α
μ (mm^−1^)	0.09
Crystal size (mm)	0.40 × 0.21 × 0.06

Data collection	
Diffractometer	Bruker APEXII CCD
Absorption correction	Multi-scan (*SADABS*; Bruker, 2003 ▸)
*T*_min_, *T*_max_	0.684, 0.746
No. of measured, independent and observed [*I* > 2σ(*I*)] reflections	22343, 4176, 2332
*R*_int_	0.058
(sin θ/λ)_max_ (Å^−1^)	0.714

Refinement	
*R*[*F* ^2^ > 2σ(*F* ^2^)], *wR*(*F* ^2^), *S*	0.062, 0.146, 1.04
No. of reflections	4176
No. of parameters	188
H-atom treatment	H atoms treated by a mixture of independent and constrained refinement
Δρ_max_, Δρ_min_ (e Å^−3^)	0.15, −0.14

Computer programs: *APEX2* (Bruker, 2003 ▸), *SAINT* (Bruker, 2003 ▸), *SHELXT* (Sheldrick, 2015*a* ▸), *SHELXL* (Sheldrick, 2015*b* ▸), *ORTEP-3 for Windows* (Farrugia, 2012 ▸), *PLATON* (Spek, 2020 ▸).

## supplementary crystallographic information

### Crystal data

**Table:**

C_16_H_14_N_2_O_2_	*F*(000) = 560
*M**_r_* = 266.29	*D*_x_ = 1.283 Mg m^−^^3^
Monoclinic, *P*2_1_/*c*	Mo *K*α radiation, λ = 0.71073 Å
*a* = 8.8644 (4) Å	Cell parameters from 3738 reflections
*b* = 19.9222 (8) Å	θ = 2.4–30.5°
*c* = 8.1736 (3) Å	µ = 0.09 mm^−^^1^
β = 107.240 (1)°	*T* = 296 K
*V* = 1378.59 (10) Å^3^	Prism, colourless
*Z* = 4	0.40 × 0.21 × 0.06 mm

### Data collection

**Table:**

Bruker APEXII CCD diffractometer	2332 reflections with *I* > 2σ(*I*)
φ and ω scans	*R*_int_ = 0.058
Absorption correction: multi-scan (SADABS; Bruker, 2003)	θ_max_ = 30.5°, θ_min_ = 2.4°
*T*_min_ = 0.684, *T*_max_ = 0.746	*h* = −12→12
22343 measured reflections	*k* = −28→28
4176 independent reflections	*l* = −11→11

### Refinement

**Table:**

Refinement on *F*^2^	0 restraints
Least-squares matrix: full	Hydrogen site location: mixed
*R*[*F*^2^ > 2σ(*F*^2^)] = 0.062	H atoms treated by a mixture of independent and constrained refinement
*wR*(*F*^2^) = 0.146	*w* = 1/[σ^2^(*F*_o_^2^) + (0.0465*P*)^2^ + 0.2174*P*] where *P* = (*F*_o_^2^ + 2*F*_c_^2^)/3
*S* = 1.03	(Δ/σ)_max_ < 0.001
4176 reflections	Δρ_max_ = 0.15 e Å^−^^3^
188 parameters	Δρ_min_ = −0.14 e Å^−^^3^

### Special details

**Table:**

Geometry. All esds (except the esd in the dihedral angle between two l.s. planes) are estimated using the full covariance matrix. The cell esds are taken into account individually in the estimation of esds in distances, angles and torsion angles; correlations between esds in cell parameters are only used when they are defined by crystal symmetry. An approximate (isotropic) treatment of cell esds is used for estimating esds involving l.s. planes.

### Fractional atomic coordinates and isotropic or equivalent isotropic displacement parameters (Å^2^)

**Table:**

	*x*	*y*	*z*	*U*_iso_*/*U*_eq_	Occ. (<1)
C1	0.7095 (2)	0.56971 (8)	0.9270 (2)	0.0519 (4)	
C2	0.76884 (18)	0.52981 (8)	0.8095 (2)	0.0482 (4)	
C3	0.92693 (18)	0.55352 (8)	0.8274 (2)	0.0493 (4)	
C4	1.0448 (2)	0.53565 (9)	0.7563 (3)	0.0624 (5)	
H4	1.028478	0.502040	0.673914	0.075*	
C5	1.1879 (2)	0.56929 (11)	0.8114 (3)	0.0728 (6)	
H5	1.268324	0.558312	0.764500	0.087*	
C6	1.2129 (2)	0.61884 (11)	0.9347 (3)	0.0749 (6)	
H6	1.310276	0.640405	0.969456	0.090*	
C7	1.0974 (2)	0.63715 (10)	1.0075 (3)	0.0673 (5)	
H7	1.114142	0.670299	1.091089	0.081*	
C8	0.95594 (19)	0.60361 (8)	0.9495 (2)	0.0538 (4)	
C9	0.4768 (2)	0.40926 (8)	0.6127 (2)	0.0530 (4)	
C10	0.5476 (2)	0.37321 (9)	0.5108 (2)	0.0603 (5)	
H10	0.648784	0.384407	0.508401	0.072*	
C11	0.4678 (3)	0.32049 (9)	0.4125 (2)	0.0702 (6)	
C12	0.3181 (3)	0.30497 (10)	0.4188 (3)	0.0779 (6)	
H12	0.263853	0.269826	0.351887	0.093*	
C13	0.2457 (2)	0.33984 (10)	0.5213 (3)	0.0693 (6)	
C14	0.3266 (2)	0.39267 (9)	0.6189 (2)	0.0598 (5)	
H14	0.280284	0.416993	0.688386	0.072*	
C15	0.5458 (3)	0.28029 (12)	0.3040 (3)	0.1039 (9)	
H15A	0.513973	0.234174	0.302284	0.156*	
H15B	0.658448	0.283384	0.351251	0.156*	
H15C	0.514651	0.297710	0.189347	0.156*	
C16	0.0820 (3)	0.32121 (12)	0.5296 (3)	0.0972 (8)	
H16D	0.052299	0.350758	0.607547	0.146*	0.67 (4)
H16E	0.082700	0.275746	0.568674	0.146*	0.67 (4)
H16F	0.007489	0.325362	0.417644	0.146*	0.67 (4)
H16A	0.013993	0.359673	0.500891	0.146*	0.33 (4)
H16B	0.088531	0.306500	0.643291	0.146*	0.33 (4)
H16C	0.039964	0.285692	0.449682	0.146*	0.33 (4)
N1	0.69683 (16)	0.48115 (7)	0.70999 (18)	0.0508 (3)	
N2	0.55439 (17)	0.46316 (7)	0.7133 (2)	0.0545 (4)	
H2	0.513 (2)	0.4843 (10)	0.792 (3)	0.077 (6)*	
O1	0.82605 (14)	0.61480 (6)	1.00954 (16)	0.0609 (3)	
O2	0.58433 (14)	0.56775 (7)	0.95705 (17)	0.0666 (4)	

### Atomic displacement parameters (Å^2^)

**Table:**

	*U*^11^	*U*^22^	*U*^33^	*U*^12^	*U*^13^	*U*^23^
C1	0.0428 (9)	0.0531 (9)	0.0561 (10)	−0.0041 (7)	0.0089 (8)	−0.0012 (8)
C2	0.0422 (9)	0.0501 (9)	0.0482 (9)	−0.0004 (7)	0.0073 (7)	0.0016 (7)
C3	0.0403 (8)	0.0503 (9)	0.0546 (10)	0.0026 (7)	0.0100 (7)	0.0083 (7)
C4	0.0525 (11)	0.0673 (11)	0.0680 (12)	0.0056 (9)	0.0186 (9)	0.0090 (9)
C5	0.0459 (11)	0.0842 (14)	0.0898 (16)	0.0066 (10)	0.0223 (11)	0.0224 (12)
C6	0.0445 (11)	0.0770 (13)	0.0958 (16)	−0.0094 (9)	0.0092 (11)	0.0179 (12)
C7	0.0512 (11)	0.0607 (11)	0.0808 (14)	−0.0092 (9)	0.0053 (10)	0.0015 (10)
C8	0.0409 (9)	0.0531 (9)	0.0630 (11)	−0.0004 (7)	0.0088 (8)	0.0055 (8)
C9	0.0536 (10)	0.0501 (9)	0.0461 (9)	−0.0056 (8)	0.0005 (8)	0.0030 (7)
C10	0.0700 (12)	0.0553 (10)	0.0511 (10)	−0.0063 (9)	0.0109 (9)	0.0003 (8)
C11	0.0950 (16)	0.0537 (11)	0.0542 (11)	−0.0090 (10)	0.0102 (11)	−0.0011 (9)
C12	0.0988 (17)	0.0575 (12)	0.0572 (12)	−0.0205 (11)	−0.0078 (12)	−0.0005 (10)
C13	0.0649 (12)	0.0635 (12)	0.0621 (12)	−0.0153 (9)	−0.0080 (10)	0.0156 (10)
C14	0.0534 (10)	0.0616 (11)	0.0553 (10)	−0.0066 (8)	0.0020 (8)	0.0065 (8)
C15	0.150 (2)	0.0746 (15)	0.0865 (17)	−0.0083 (15)	0.0348 (17)	−0.0243 (13)
C16	0.0724 (15)	0.0949 (17)	0.1019 (18)	−0.0327 (12)	−0.0085 (13)	0.0192 (14)
N1	0.0466 (8)	0.0525 (8)	0.0497 (8)	−0.0017 (6)	0.0086 (6)	0.0028 (6)
N2	0.0475 (8)	0.0574 (9)	0.0553 (9)	−0.0059 (7)	0.0100 (7)	−0.0065 (7)
O1	0.0496 (7)	0.0610 (7)	0.0690 (8)	−0.0061 (6)	0.0127 (6)	−0.0130 (6)
O2	0.0473 (7)	0.0790 (9)	0.0757 (9)	−0.0041 (6)	0.0213 (6)	−0.0101 (7)

### Geometric parameters (Å, º)

**Table:**

C1—O2	1.2064 (19)	C10—H10	0.9300
C1—O1	1.3842 (19)	C11—C12	1.378 (3)
C1—C2	1.459 (2)	C11—C15	1.506 (3)
C2—N1	1.304 (2)	C12—C13	1.384 (3)
C2—C3	1.445 (2)	C12—H12	0.9300
C3—C8	1.380 (2)	C13—C14	1.385 (2)
C3—C4	1.385 (2)	C13—C16	1.519 (3)
C4—C5	1.386 (3)	C14—H14	0.9300
C4—H4	0.9300	C15—H15A	0.9600
C5—C6	1.381 (3)	C15—H15B	0.9600
C5—H5	0.9300	C15—H15C	0.9600
C6—C7	1.377 (3)	C16—H16D	0.9600
C6—H6	0.9300	C16—H16E	0.9600
C7—C8	1.375 (2)	C16—H16F	0.9600
C7—H7	0.9300	C16—H16A	0.9600
C8—O1	1.397 (2)	C16—H16B	0.9600
C9—C10	1.383 (2)	C16—H16C	0.9600
C9—C14	1.387 (2)	N1—N2	1.3206 (19)
C9—N2	1.402 (2)	N2—H2	0.93 (2)
C10—C11	1.383 (2)		
			
O2—C1—O1	121.34 (16)	C11—C12—C13	122.29 (18)
O2—C1—C2	130.48 (16)	C11—C12—H12	118.9
O1—C1—C2	108.18 (14)	C13—C12—H12	118.9
N1—C2—C3	125.97 (16)	C12—C13—C14	118.4 (2)
N1—C2—C1	127.59 (15)	C12—C13—C16	121.7 (2)
C3—C2—C1	106.43 (14)	C14—C13—C16	119.9 (2)
C8—C3—C4	119.17 (16)	C13—C14—C9	119.93 (19)
C8—C3—C2	106.10 (15)	C13—C14—H14	120.0
C4—C3—C2	134.70 (17)	C9—C14—H14	120.0
C3—C4—C5	118.10 (19)	C11—C15—H15A	109.5
C3—C4—H4	121.0	C11—C15—H15B	109.5
C5—C4—H4	121.0	H15A—C15—H15B	109.5
C6—C5—C4	121.07 (19)	C11—C15—H15C	109.5
C6—C5—H5	119.5	H15A—C15—H15C	109.5
C4—C5—H5	119.5	H15B—C15—H15C	109.5
C7—C6—C5	121.71 (19)	C13—C16—H16D	109.5
C7—C6—H6	119.1	C13—C16—H16E	109.5
C5—C6—H6	119.1	H16D—C16—H16E	109.5
C8—C7—C6	116.17 (19)	C13—C16—H16F	109.5
C8—C7—H7	121.9	H16D—C16—H16F	109.5
C6—C7—H7	121.9	H16E—C16—H16F	109.5
C7—C8—C3	123.77 (18)	C13—C16—H16A	109.5
C7—C8—O1	124.32 (17)	C13—C16—H16B	109.5
C3—C8—O1	111.89 (14)	H16A—C16—H16B	109.5
C10—C9—C14	120.63 (16)	C13—C16—H16C	109.5
C10—C9—N2	121.31 (16)	H16A—C16—H16C	109.5
C14—C9—N2	118.05 (17)	H16B—C16—H16C	109.5
C11—C10—C9	119.94 (19)	C2—N1—N2	118.89 (15)
C11—C10—H10	120.0	N1—N2—C9	120.08 (15)
C9—C10—H10	120.0	N1—N2—H2	117.9 (13)
C12—C11—C10	118.8 (2)	C9—N2—H2	121.7 (13)
C12—C11—C15	121.18 (19)	C1—O1—C8	107.39 (13)
C10—C11—C15	120.0 (2)		
			
O2—C1—C2—N1	−1.0 (3)	N2—C9—C10—C11	−179.74 (16)
O1—C1—C2—N1	178.73 (15)	C9—C10—C11—C12	−0.1 (3)
O2—C1—C2—C3	−179.52 (18)	C9—C10—C11—C15	−178.83 (18)
O1—C1—C2—C3	0.23 (17)	C10—C11—C12—C13	−0.7 (3)
N1—C2—C3—C8	−178.02 (16)	C15—C11—C12—C13	178.06 (19)
C1—C2—C3—C8	0.52 (17)	C11—C12—C13—C14	0.8 (3)
N1—C2—C3—C4	−0.4 (3)	C11—C12—C13—C16	−178.75 (19)
C1—C2—C3—C4	178.17 (19)	C12—C13—C14—C9	−0.3 (3)
C8—C3—C4—C5	−0.1 (3)	C16—C13—C14—C9	179.33 (17)
C2—C3—C4—C5	−177.49 (18)	C10—C9—C14—C13	−0.5 (3)
C3—C4—C5—C6	0.5 (3)	N2—C9—C14—C13	179.91 (15)
C4—C5—C6—C7	−0.3 (3)	C3—C2—N1—N2	176.94 (15)
C5—C6—C7—C8	−0.4 (3)	C1—C2—N1—N2	−1.3 (2)
C6—C7—C8—C3	0.9 (3)	C2—N1—N2—C9	−177.48 (14)
C6—C7—C8—O1	179.25 (16)	C10—C9—N2—N1	1.1 (2)
C4—C3—C8—C7	−0.7 (3)	C14—C9—N2—N1	−179.26 (15)
C2—C3—C8—C7	177.41 (16)	O2—C1—O1—C8	178.89 (16)
C4—C3—C8—O1	−179.19 (14)	C2—C1—O1—C8	−0.88 (17)
C2—C3—C8—O1	−1.11 (19)	C7—C8—O1—C1	−177.24 (17)
C14—C9—C10—C11	0.6 (3)	C3—C8—O1—C1	1.27 (19)

### Hydrogen-bond geometry (Å, º)

*Cg*2 and *Cg*3 are the centroids of the C3–C8 and C9–C14 rings, respectively.

**Table:**

*D*—H···*A*	*D*—H	H···*A*	*D*···*A*	*D*—H···*A*
N2—H2···O2	0.93 (2)	2.12 (2)	2.840 (2)	133.8 (16)
C15—H15*A*···*Cg*3^i^	0.96	2.90	3.591 (3)	130
C16—H16*F*···*Cg*2^ii^	0.96	2.92	3.715 (3)	141

Symmetry codes: (i) *x*, −*y*−1/2, *z*−3/2; (ii) −*x*+1, −*y*+1, −*z*+1.
